# Emergency Department disposition decisions and associated mortality and costs in ICU patients with suspected infection

**DOI:** 10.1186/s13054-018-2096-8

**Published:** 2018-07-06

**Authors:** Shannon M. Fernando, Bram Rochwerg, Peter M. Reardon, Kednapa Thavorn, Andrew J. E. Seely, Jeffrey J. Perry, Douglas P. Barnaby, Peter Tanuseputro, Kwadwo Kyeremanteng

**Affiliations:** 1 Division of Critical Care, Department of Medicine, University of Ottawa, Ottawa, ON Canada; 2 Department of Emergency Medicine, University of Ottawa, Ottawa, ON Canada; 3 Division of Critical Care, Department of Medicine, McMaster University, Hamilton, ON Canada; 4 Department of Health Research Methods, Evidence, and Impact, McMaster University, Hamilton, ON Canada; 5 School of Epidemiology and Public Health, University of Ottawa, Ottawa, ON Canada; 6 Clinical Epidemiology Program, Ottawa Hospital Research Institute, Ottawa, ON Canada; 7 Department of Surgery, University of Ottawa, Ottawa, ON Canada; 8 Department of Emergency Medicine, Albert Einstein College of Medicine, Bronx, NY USA; 9 Bruyere Research Institute, Ottawa, ON Canada; 10 Division of Palliative Care, Department of Medicine, University of Ottawa, Ottawa, ON Canada

**Keywords:** Sepsis, Emergency department, Infectious diseases, Disposition, Hospital costs

## Abstract

**Background:**

Following emergency department (ED) assessment, patients with infection may be directly admitted to the intensive care unit (ICU) or alternatively admitted to hospital wards or sent home. Those admitted to the hospital wards or sent home may experience future deterioration necessitating ICU admission.

**Methods:**

We used a prospectively collected registry from two hospitals within a single tertiary care hospital network between 2011 and 2014. Patient information, outcomes, and costs were stored in the hospital data warehouse. Patients were categorized into three groups: (1) admitted directly from the ED to the ICU; (2) initially admitted to the hospital wards, with ICU admission within 72 hours of initial presentation; or (3) sent home from the ED, with ICU admission within 72 hours of initial presentation. Using multivariable logistic regression, we sought to compare outcomes and total costs between groups. Total costs were evaluated using a generalized linear model.

**Results:**

A total of 657 patients were included; of these, 338 (51.4%) were admitted directly from the ED to the ICU, 246 (37.4%) were initially admitted to the wards and then to the ICU, and 73 (11.1%) were initially sent home and then admitted to the ICU. In-hospital mortality was lowest among patients admitted directly to the ICU (29.5%), as compared with patients admitted to the ICU from wards (42.7%) or home (61.6%) (*P* < 0.001). As compared with direct ICU admission, disposition to the ward was associated with an adjusted OR of 1.75 (95% CI, 1.22–2.50; *P* < 0.01) for mortality, and disposition home was associated with an adjusted OR of 4.02 (95% CI, 2.32–6.98). Mean total costs were lowest among patients directly admitted to the ICU ($26,748), as compared with those admitted from the wards ($107,315) and those initially sent home ($71,492) (*P* < 0.001). Cost per survivor was lower among patients directly admitted to the ICU ($37,986) than either those initially admitted to the wards ($187,230) or those sent home ($186,390) (*P* < 0.001).

**Conclusions:**

In comparison with direct admission to the ICU, patients with suspected infection admitted to the ICU who have previously been discharged home or admitted to the ward are associated with higher in-hospital mortality and costs.

**Electronic supplementary material:**

The online version of this article (10.1186/s13054-018-2096-8) contains supplementary material, which is available to authorized users.

## Background

Sepsis is a major cause of morbidity and mortality, with approximately 19 million cases per year worldwide and resulting in 5.3 million deaths [1]. In the emergency department (ED), patients presenting with suspected infection represent an enormous burden, with an estimated 850,000 visits per year in the United States alone [2]. ED physicians have traditionally used the systemic inflammatory response syndrome (SIRS) criteria for screening patients with suspected infection [3], with presence of two or more of the criteria being indicative of sepsis [4]. Rapid initiation of treatment in these patients has improved outcomes, whereas delays in treatment are associated with increased mortality [5, 6].

Aside from sepsis recognition and treatment initiation, in order to appropriately risk-stratify and optimize disposition, ED clinicians must also be able to predict short-term deterioration in patients presenting with infection [7]. This includes determining whether the patient requires treatment and monitoring in the intensive care unit (ICU), is appropriate for management on the hospital wards, or is well enough for discharge home.

Patients discharged home or admitted to the hospital wards may still require ICU admission if they experience future clinical deterioration. Prediction of short-term deterioration is challenging because it must account for a variety of important patient- and disease-specific factors [8]. Previous studies have demonstrated that patients with critical illness admitted to the ICU from the hospital wards have worse outcomes than those admitted directly from the ED, independent of disease severity [9]. However, these findings were confounded by significant clinical heterogeneity among patients admitted from the wards, with many having been admitted for weeks (and sometimes months) prior to ICU admission and some being transferred directly from other hospitals without an index ED visit. Similarly, patients with community-acquired pneumonia who were initially admitted to the hospital wards but later required ICU admission within 72 hours were found to have increased mortality compared with those admitted directly to the ICU from the ED [10]. Whether these findings are generalizable to patients with other sources of infection or to those who are initially discharged to home and then require ICU admission is unclear. Finally, little is known regarding the impact of delayed ICU disposition on overall hospital costs, because patients with infection account for a significant proportion of hospital spending [11]. In the present study, we sought to investigate the outcomes and associated costs of patients directly admitted from the ED to the ICU with a diagnosis of suspected infection (including those with and without sepsis) and to compare them with patients initially admitted to the hospital wards or initially discharged to home but requiring ICU admission within 72 hours.

## Methods

Ethics approval for this study was obtained from The Ottawa Health Science Network Research Ethics Board.

### Study design, setting, and subjects

We studied patients at two individual academic hospitals within The Ottawa Hospital network (Ottawa, ON, Canada). The combined network has 1163 beds and handles over 160,000 emergency visits annually. Each hospital has a combined medical-surgical ICU, with 28 ICU beds at each, and approximately 2500 combined ICU admissions per year. If an ED physician believes a patient requires ICU admission, the ICU is consulted. Decisions related to ICU or ward admission are made by the admitting service in discussion and collaboration with the referring ED physician. There are no electronic or manual screening tools used for early detection of deterioration on the hospital wards. We conducted a retrospective analysis of prospectively collected data from The Ottawa Hospital Data Warehouse, a health administrative database that has been used widely in previous research [12–15]. Data quality assessments were performed during development and are executed routinely as new data are included. Quality assurance initiatives are conducted regularly to ensure completeness and accuracy.

We included all patients ≥ 18 years of age admitted to one of the participating ICUs between 2011 and 2014 and with an index ED encounter diagnosis of suspected infection, as well as an admission or discharge diagnosis of suspected infection, using International Classification of Diseases, Tenth Revision, Canada (ICD-CA, version 10), codes (Additional file 1: Table S1). Charts of patients admitted to the ICU with a diagnosis of suspected infection (including those with and without sepsis) were reviewed by an investigator blinded to patient outcomes in order to ensure that both the ED encounter diagnosis and the ICU admission diagnosis were consistent with the stored ICD-CA code. Patients initially diagnosed as having suspected infection by the ED physician but who were admitted to the ICU for a non-infection-related diagnosis were excluded. All patients had to have been admitted to the ICU within 72 hours of their index ED presentation. We excluded patients transferred directly to the ICU from a peripheral hospital, because ED data could not be obtained, as well as patients without an index ED visit 72 hours prior to ICU admission (e.g., those admitted for elective surgery but ultimately requiring ICU admission). We also excluded patients whose index ED visit was deemed to be unrelated to infection and those who left the index ED encounter against medical advice. Importantly, we excluded patients who, at the time of ED arrival, had a goals-of-care status precluding ICU admission. Patients were categorized into three groups: (1) admitted directly to the ICU from the ED; (2) admitted to wards from the ED, with subsequent deterioration requiring ICU admission within 72 hours; or (3) sent home from the ED, with subsequent deterioration requiring ICU admission within 72 hours.

### Data collection

We obtained all data from The Ottawa Hospital Data Warehouse. For each patient, we abstracted basic demographic data, comorbidities, and Elixhauser comorbidity scores [16]. ED and ICU data collected included initial vital signs, initial laboratory values, and Acute Physiology and Chronic Health Evaluation II (APACHE II) score [17], based on initial ED and ICU values. Partial pressure of arterial oxygen values were estimated from peripheral capillary oxygen saturation values as described previously [18, 19]. Data were collected from admission until either the point of discharge from the hospital or in-hospital death. This included ICU length of stay (LOS), hospital LOS, and final disposition status. Bacteremia was defined as bacterial growth in at least two blood cultures from the same patient, with confirmation from a microbiologist that the organism did not represent a contaminant. This definition was deliberately more restrictive than what may be used in clinical practice, in order to minimize the influence of contaminants. Septic shock was defined as initiation of vasopressors to maintain a mean arterial pressure ≥ 65 mmHg, coupled with a serum lactate > 2.0 mmol/L, as defined by the Third International Consensus Definitions for Sepsis and Septic Shock (Sepsis-3) [20, 21]. Data on the use of mechanical ventilation, renal replacement therapy, and vasoactive medications were not available.

We determined patient costs using the case-costing system of The Ottawa Hospital Data Warehouse [13, 14]. Total costs include both direct and indirect costs from a hospital perspective. Direct costs refer to all expenses to the hospital with fee codes linked to the patient chart. Included among direct costs are salaries and benefits for unit producing and management staff, equipment, and screening and procedure materials, but they do not include physician remuneration. Indirect costs refer to any overhead operational fees associated with the service being provided to the patient, such as the cost of the room they occupy. Total costs are divided by the number of patients surviving to hospital discharge in order to determine the cost per survivor. The Ottawa Hospital uses a standardized case-costing methodology that was developed by the Ontario Case Costing Initiative and is based on the Canadian Institute for Health Information Management Information Systems guidelines [22]. Costs were then indexed (to 2018 Canadian dollars) using consumer price indices [13, 23, 24].

The primary outcome was in-hospital mortality. Secondary outcomes included ICU LOS, total hospital LOS, total costs, cost per day, and cost per survivor.

### Statistical analysis

All statistical analyses were performed with commercially available statistical software packages (R version 3.3.3, R Foundation for Statistical Computing; IBM SPSS Statistics version 24.0, IBM, Armonk, NY, USA). Data are presented as mean values with standard deviation (SD) or medians with interquartile ranges (IQRs), when appropriate. One-way analysis of variance (ANOVA) (for parametric values), the Kruskal-Wallis test (for nonparametric values), and the χ^2^ test (for categorical values) were used to test comparisons. If ANOVA or the Kruskal-Wallis test indicated a statistically significant difference, pairwise comparisons with either Student’s *t* test (parametric values) or the Mann-Whitney *U* test (nonparametric values) were performed to determine between-group differences. In evaluating the outcome of in-hospital mortality, we used multivariable logistic regression modeling to adjust for potential confounders, including age, sex, and Elixhauser comorbidity index. Variation in total costs was assessed using a multivariable generalized linear model to generate cost coefficients and 95% CIs. A gamma distribution was applied. Generalized linear models are the recommended methodology for modeling the impact of covariates in cost analyses of health services, because they can account for significant skew without the need for transformation [25, 26]. A *P* value ≤ 0.05 was considered statistically significant.

## Results

**Fig. 1:**
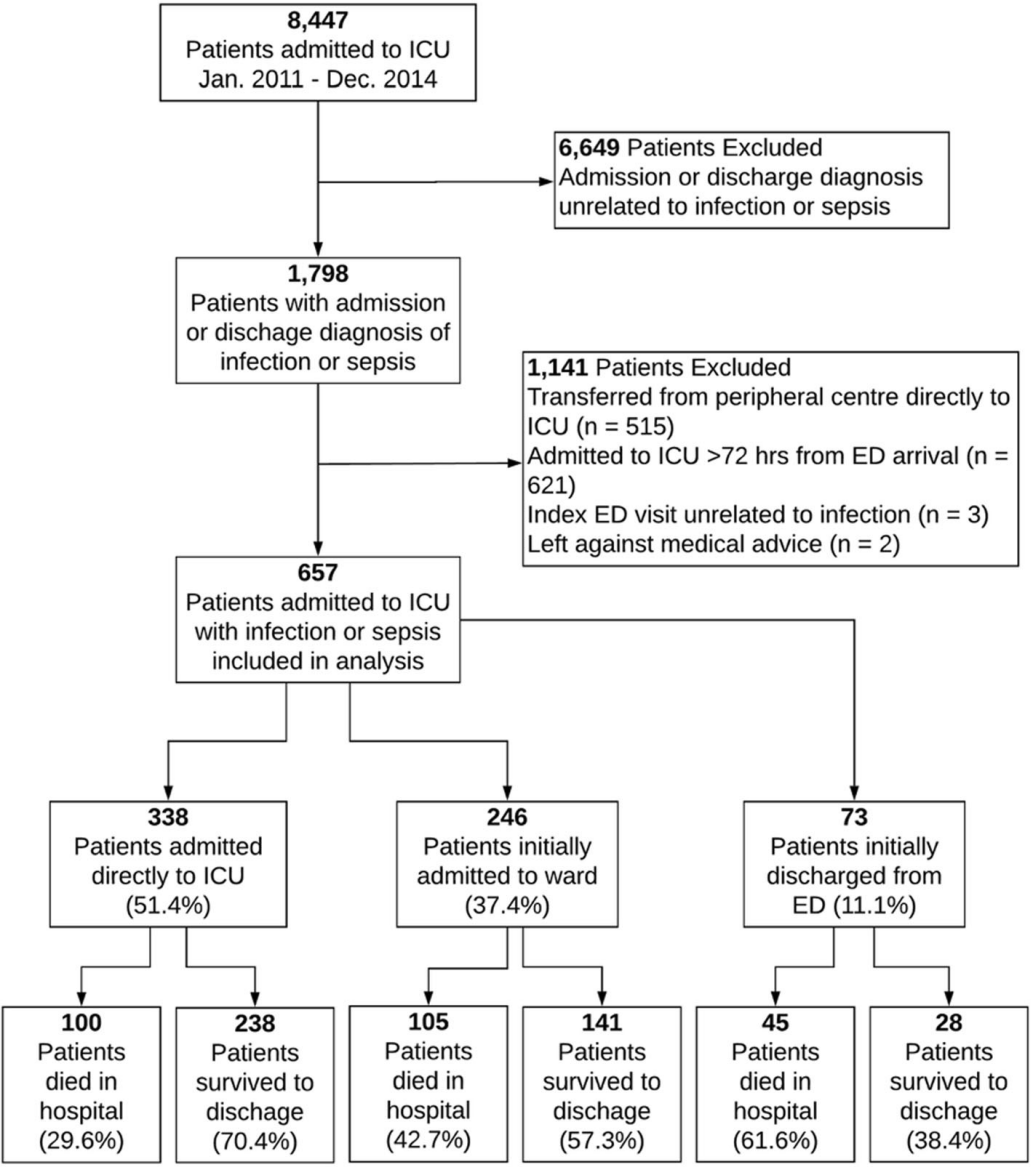
Patient flow diagram. *ICU* Intensive care unit; *ED* Emergency department

From 2011 to 2014, 8447 patients were admitted to one of the participating ICUs (Fig. 1). Of these, 1798 (21.3%) patients had an admission or discharge diagnosis of infection, and 515 patients (28.6%) were transferred directly to the ICU from a periphery hospital and excluded. Another 621 (34.5%) were excluded because they were admitted to the ICU more than 72 hours after the index ED presentation.

We included the remaining 657 patients in our analysis. Of these, 338 (51.4%) were admitted directly from the ED to the ICU, 246 patients (37.4%) were admitted from the ED to the wards followed by ICU admission within 72 hours of arrival at their index ED presentation, and 73 patients (11.1%) were discharged to home from the ED but returned to the hospital and were admitted to the ICU within 72 hours of arrival at their index ED presentation.

Table 1 displays the characteristics of included ICU patients with infection, categorized by disposition. Mean APACHE II score at the time of index ED presentation was higher for patients admitted directly to the ICU (13.5, SD 3.6) than for those initially admitted to the wards (12.4, SD 3.7) or discharged to home (7.7, SD 2.6) (*P* < 0.001). Mean APACHE II score at the time of ICU admission was highest among those initially discharged to home but who later required ICU admission (17.4, SD 4.5) (*P* < 0.001).

**Table 1:** Characteristics of patients with suspected infection admitted to the intensive care unit

Characteristic	Direct to ICU (*n* = 338)	Ward (*n* = 246)	Home (*n* = 73)	*P* value
Age, years, mean (SD)	64.4 (16.4)	64.3 (14.1)	63.1 (16.0)	0.61
Male sex, *n* (%)	169 (50.0)	155 (63.0)	36 (49.3)	< 0.01^a,b^
Comorbidities, *n* (%)				
Congestive heart failure	22 (6.5)	32 (13.0)	8 (11.0)	0.03^a^
Arrhythmia	44 (13.0)	46 (18.7)	12 (16.4)	0.17
Valvular disease	7 (2.1)	6 (2.4)	0 (0)	0.42
Peripheral vascular disease	3 (0.9)	11 (4.5)	3 (4.1)	0.02^a,c^
Hypertension	54 (16.0)	62 (25.2)	16 (21.9)	0.02^a^
Chronic obstructive pulmonary disease	30 (8.9)	33 (13.4)	9 (12.3)	0.95
Diabetes mellitus	93 (27.5)	80 (32.5)	30 (41.1)	0.06
Renal failure	25 (7.4)	25 (10.2)	6 (8.2)	0.40
Liver disease	21 (6.2)	25 (10.2)	6 (8.2)	0.22
Metastatic cancer	24 (7.1)	15 (6.1)	5 (6.8)	0.89
Elixhauser comorbidity score, mean (SD)	5.2 (4.1)	7.2 (6.8)	6.9 (5.1)	< 0.01^a,c^
ED APACHE II score, mean (SD)	13.5 (3.6)	12.4 (3.7)	7.7 (2.6)	< 0.001^a,b,c^
ICU APACHE II score, mean (SD)	13.6 (3.6)	15.6 (3.9)	17.4 (4.5)	< 0.001^a,b,c^
Suspected source of infection, *n* (%)				0.87
Pulmonary	170 (50.3)	116 (47.2)	29 (39.7)	
Gastrointestinal	93 (27.5)	72 (29.3)	24 (32.9)	
Urinary tract	51 (15.1)	40 (16.3)	12 (16.4)	
Skin/soft tissue infection	8 (2.4)	5 (2.0)	4 (5.5)	
Central nervous system	7 (2.1)	5 (2.0)	2 (2.7)	
Other/unknown	9 (2.7)	8 (3.3)	2 (2.7)	
Bacteremia, *n* (%)	74 (21.9)	50 (20.3)	15 (20.5)	0.89
Septic shock in ED, *n* (%)	220 (65.1)	0 (0.0)	0 (0.0)	< 0.001^a,c^
Initial vital signs				
Systolic blood pressure, mmHg, mean (SD)	105 (24)	121 (29)	124 (32)	< 0.001^a,c^
Diastolic blood pressure, mmHg, mean (SD)	61 (18)	71 (15)	74 (18)	< 0.001^a,c^
Heart rate, beats/min, mean (SD)	105 (33)	92 (30)	90 (31)	< 0.001^a,c^
Temperature, °C, mean (SD)	37.6 (0.8)	36.8 (0.7)	36.8 (0.7)	< 0.01^a,c^
Oxygen saturation, %, median (IQR)	90 (5)	94 (5)	94 (5)	< 0.01^a,c^
Initial laboratory values				
White blood cell count, × 10^9^/L, median (IQR)	8.7 (5.9–10.9)	7.9 (5.6–10.2)	7.9 (5.5–10.5)	0.10
Hemoglobin, g/L, mean (SD)	109 (21)	110 (19)	111 (19)	0.65
Platelets, × 10^9^/L, mean (SD)	280 (103)	288 (98)	278 (116)	0.13
Sodium, mmol/L, mean (SD)	140 (5)	140 (5)	141 (6)	0.28
Potassium, mmol/L, mean (SD)	4.3 (0.5)	4.3 (0.6)	4.2 (0.7)	0.40
Creatinine, μmol/L, median (IQR)	110 (93–129)	102 (81–107)	93 (82–98)	< 0.001^a,b,c^
Urea, mmol/L, median (IQR)	8.3 (4.9–17.9)	6.6 (4.5–11.8)	4.8 (3.7–5.8)	< 0.001^a,b,c^
Lactate, mmol/L, median (IQR)	3.2 (2.3–4.1)	3.2 (2.6–3.6)	2.3 (1.7–2.7)	< 0.001^b,c^
Albumin, g/L, mean (SD)	27.1 (6.8)	27.3 (6.9)	27.3 (6.8)	0.34
INR, median (IQR)	1.2 (1.1–1.4)	1.2 (1.1–1.4)	1.2 (1.1–1.3)	0.49

*Abbreviations: ICU* Intensive care unit, *ED* Emergency department, *APACHE II* Acute Physiology and Chronic Health Evaluation II, *IQR* Interquartile range, INR International normalized ratio

Characteristics of ICU patients with suspected infection and index visit to ED within 72 hours of ICU admission. One-way analysis of variance was used to compare mean values, and the Kruskal-Wallis test was used to compare median values between groups. The χ^2^ test was used to compare categorical variables between groups. *P* values are displayed. Pairwise comparisons were performed using Student’s *t* test (parametric values) and the Mann-Whitney *U* test (nonparametric values)

^a^Statistically significant difference (i.e., *P* < 0.05) between patients admitted directly to the ICU and those admitted to the ward

^b^Statistically significant difference (i.e., *P* < 0.05) between patients admitted to the ward and those initially sent home

^c^Statistically significant difference (i.e., *P* < 0.05) between patients admitted directly to the ICU and those initially sent home

Among initial vital signs at the time of ED arrival, patients admitted directly to the ICU had significantly lower mean systolic blood pressure, diastolic blood pressure, and oxygen saturation, as well as higher mean heart rate and body temperature, than patients initially admitted to the wards or discharged to home (all *P* values < 0.01). Regarding initial laboratory values in the ED, patients admitted directly to the ICU had significantly higher serum lactate, creatinine, and urea (all *P* values < 0.001). Treatments delivered to patients in the ED are displayed in Table 2.

**Table 2:** Emergency department interventions

Outcome	Direct to ICU (*n* = 338)	Ward (*n* = 246)	Home (*n* = 73)	*P* value
Fluids, *n* (%)				< 0.001^a,b,c^
None	10 (3.0)	83 (33.7)	56 (76.7)	
≤ 1 L	126 (37.2)	140 (56.9)	12 (16.4)	
> 1 L	202 (59.8)	23 (9.3)	5 (6.8)	
Antimicrobial therapy, *n* (%)				< 0.001^a,b,c^
None	5 (1.5)	12 (4.9)	7 (9.6)	
Oral	4 (1.1)	32 (13.0)	62 (84.9)	
Parenteral	329 (97.3)	202 (82.1)	4 (5.5)	
Corticosteroid therapy, *n* (%)				< 0.001^a,b,c^
None	60 (17.8)	174 (70.7)	65 (89.1)	
Oral	51 (15.1)	60 (24.4)	8 (11.0)	
Parenteral	227 (67.2)	12 (4.9)	0 (0)	
Initiation of vasopressors, *n* (%)	301 (89.1)	0 (0)	0 (0)	< 0.001^a,b^
Mechanical ventilation, *n* (%)	220 (65.1)	0 (0)	0 (0)	< 0.001^a,b^

*ICU* Intensive care unit

Pairwise comparisons were performed using χ^2^ test

^a^Statistically significant difference (i.e., *P* < 0.05) between patients admitted directly to the ICU and those admitted to the ward

^b^Statistically significant difference (i.e., *P* < 0.05) between patients admitted directly to the ICU and those initially sent home

^c^Statistically significant difference (i.e., *P* < 0.05) between patients admitted to the ward and those initially sent home

Patient outcomes are depicted in Table 3. Crude in-hospital mortality was lowest among patients admitted directly to the ICU (29.5%), as compared with patients initially admitted to the ward (42.7%) and those initially discharged to home (61.6%) (*P* < 0.001). Median ICU LOS was also shortest among patients admitted directly to the ICU (4 days, IQR 2–7 days), as compared with those initially admitted to the ward (12 days, IQR 7–20 days) and those initially discharged to home (8 days, IQR 2.5–14 days). When comparing the disposition of patients surviving to hospital discharge, patients admitted directly to the ICU had a higher proportion of survivors discharged to home, as compared with those initially admitted to the wards or discharged to home (*P* < 0.001). The adjusted OR for in-hospital mortality for patients initially discharged home (as compared with those initially admitted to the ICU) was 4.02 (95% CI 2.32–6.98) (Additional file 2: Table S2). Similarly, adjusted OR for in-hospital mortality for patients admitted to the wards (as compared with those initially admitted to the ICU) was 1.75 (95% CI 1.22–2.50, *P* < 0.01).

**Table 3:** Outcomes of patients admitted to the intensive care unit with suspected infection

Outcome	Direct to ICU (*n* = 338)	Ward (*n* = 246)	Home (*n* = 73)	*P* value
In-hospital mortality, *n* (%)	100 (29.5)	105 (42.7)	45 (61.6)	< 0.001^a,b,c^
ICU length of stay, days, median (IQR)	4 (2–7)	12 (7–20)	8 (2.5–14)	< 0.001^a,b,c^
ICU length of stay among survivors to hospital discharge, days, median (IQR)	4 (2–7)	13 (7–20)	8.5 (3–21)	< 0.001^a,b,c^
ICU length of stay among deceased in-hospital, days, median (IQR)	3 (1–6.8)	20 (6–20)	7 (2–12)	< 0.001^a,b,c^
Hospital length of stay, days, median (IQR)	7.5 (3–13.3)	26 (14–46)	14 (5–32)	< 0.001^a,b,c^
Hospital length of stay among survivors to hospital discharge, days, median (IQR)	10 (6–15)	32 (17–61.5)	33.5 (23.8–59)	< 0.001^a,b^
Hospital length of stay among deceased in hospital, days, median (IQR)	2 (1–4.8)	20 (9–33)	6 (3–12.5)	< 0.001^a,b,c^
Disposition, *n* (%)				< 0.001^a,b,c^
Home	187 (55.3)	83 (33.7)	13 (17.8)	
Acute care facility transfer	20 (5.9)	14 (5.7)	3 (4.1)	
Long-term care facility transfer	31 (9.2)	44 (17.9)	12 (16.4)	

*ICU* Intensive care unit

Outcomes of ICU patients with suspected infection and index ED visit within 72 hours of ICU admission, categorized by initial disposition destination. The χ^2^ test was used to compare categorical variables. The Kruskal-Wallis test was used to compare median values between groups, and resultant *P* values are listed. Pairwise comparisons were performed using the Mann-Whitney *U* test

^a^Statistically significant difference (i.e., *P* < 0.05) between patients admitted directly to the ICU and those admitted to the ward

^b^Statistically significant difference (i.e., *P* < 0.05) between patients admitted directly to the ICU and those initially sent home

^c^Statistically significant difference (i.e., *P* < 0.05) between patients admitted to the ward and those initially sent home

Costs of included patients are displayed in Table 4. Mean total costs of patients admitted directly to the ICU ($26,748, SD $24,706) were significantly lower than those of patients initially admitted to the ward ($107,315, SD $92,887) and those discharged to home ($71,492, SD $65,149) (*P* < 0.001). Mean cost per day was also lowest among patients initially admitted to the ICU ($3443, SD $2238), as compared with those initially discharged to home ($4071, SD $2452) (*P* = 0.04). Cost per survivor was also lowest among patients admitted directly to the ICU ($37,986 [95% CI: $37,962-38,012]), as compared with patients initially admitted to the ward ($187,230, 95% CI $187,159–$187,302]) and those initially discharged to home ($186,390, 95% CI $186,230–$187,550). Differences in total cost between patients initially admitted to the ICU and those admitted to the wards or discharged to home were also found when evaluating the generalized linear model (Additional file 3: Table S3), with direct ICU disposition having a statistically significant impact on overall costs (*P* < 0.001).

**Table 4:** Costs of patients admitted to the intensive care unit with suspected infection

	Direct to ICU (*n* = 338)	Ward (*n* = 246)	Home (*n* = 73)	*P* value
Overall costs, Can$				
Total direct costs, mean (SD)	20,196 (16,258)	81,068 (72,858)	54,206 (44,805)	< 0.001^a,b,c^
Total indirect costs, mean (SD)	6552 (5475)	26,247 (20,136)	17,286 (10,455)	< 0.001^a,b,c^
Total cost, mean (SD)	26,748 (24,706)	107,315 (92,887)	71,492 (65,149)	< 0.001^a,b,c^
Cost/day, mean (SD)	3443 (2238)	3504 (2150)	4071 (2452)	0.04^b^
Cost/survivor (95% CI)	37,986 (37,962–38,012)	187,230 (187,159–187,302)	186,390 (186,230–187,550)	< 0.001^a,b^
Cost/survivor discharged home (95% CI)	48,347 (48,316–48,379)	318,067 (317,945–318,188)	401,454 (401,110–401,799)	< 0.001^a,b,c^
Cost allocation, Can$, mean (SD)				
Food services	1054 (956)	1939 (1490)	482 (382)	< 0.001^a,b,c^
ICU	12,283 (10,030)	41,919 (38,276)	35,247 (33,928)	< 0.001^a,b^
Laboratory tests	1464 (917)	5302 (4772)	3300 (3274)	< 0.001^a,b^
Imaging	839 (665)	3066 (2636)	1853 (1614)	< 0.001^a,b^
Pharmacy	1207 (843)	7229 (6468)	3938 (2927)	< 0.001^a,b^
Health professional services (nonphysician)	1157 (954)	3852 (3308)	1958 (1425)	< 0.001^a^
Nursing	5818 (4429)	26,801 (25,994)	12,677 (10,244)	< 0.001^a,b,c^

*ICU* Intensive care unit, *IQR* Interquartile range

Costs of ICU patients with suspected infection and index visit to ED within 72 hours of ICU admission, categorized by disposition destination, are shown. ICU costs refer to all costs not included in other subcategories, and include costs of mechanical ventilation, renal replacement therapy, and vasoactive medications. All values are in Canadian dollars. Analysis of variance was used to compare mean values between groups, and resultant *P* values are listed. Pairwise comparisons were performed using Student’s *t* test

^a^Statistically significant difference (i.e., *P* < 0.05) between patients admitted directly to the ICU and those admitted to the ward

^b^Statistically significant difference (i.e., *P* < 0.05) between patients admitted directly to the ICU and those initially sent home

^c^Statistically significant difference (i.e., *P* < 0.05) between patients admitted to the ward and those initially sent home

## Discussion

We found that patients with suspected infection directly admitted from the ED to the ICU had lower mortality, ICU LOS, total hospital LOS, and total costs than those initially admitted to the hospital wards or sent home but who ultimately needed ICU admission. Our findings demonstrate worse outcomes and higher costs in ED patients with unpredicted short-term deterioration (i.e., those initially admitted to the ward or sent home who ultimately ended up in the ICU within 72 hours), and they highlight the importance of optimization of disposition in ED patients presenting with infection and at risk of future deterioration.

In our cohort, 48.6% of patients ultimately requiring the ICU within 72 hours of ED arrival were either admitted to the hospital wards or discharged to home. This prevalence is similar to other reports [9]. The process of optimizing disposition is a combined effort including ED clinicians and admitting consultants, and decisions related to disposition are multifactorial. These findings may be partly related to impaired detection of sepsis. Although all patients were diagnosed with infection, it is possible that the ED physicians did not identify more subtle evidence of sepsis. There is existing evidence suggesting that ED physicians significantly underrecognize sepsis among patients presenting with infection [27, 28].

More important than detection, there may be misjudgments in risk stratification and prognostication. The fact that only roughly half of patients ultimately requiring ICU admission are identified during their initial ED visit highlights the difficulty of predicting future short-term deterioration in patients with infection and sepsis. At the time of ED disposition, there is limited information available to both ED physicians and consultants. For example, the prevalence of bacteremia was similar between patients admitted directly to the ICU and those admitted to the ward or sent home, but blood culture results are rarely available at the time of ED disposition. Currently, most protocols for care of patients with infection (and suspected sepsis) are predicated on the SIRS criteria [3] because use of such protocols has been associated with improved outcomes [6]. The sensitivity of the SIRS criteria for prediction of mortality in ED patients with suspected infection has been estimated at approximately 84%, suggesting that they may still miss a proportion of patients at risk of death [29]. Additionally, the SIRS criteria have been found to be nonspecific because they are seen in a variety of conditions unrelated to infection [30], with estimated specificity of 30% in ED patients [29].

In our cohort, patients admitted to the ICU directly seemed to have a greater number of vital sign abnormalities consistent with the SIRS criteria (such as higher heart rates and body temperatures), suggesting that ED and consulting clinicians may have focused on such parameters when determining disposition. The shortcomings of the SIRS criteria as a risk stratification tool suggest that alternative criteria may be helpful. The proposed quick Sequential (Sepsis-related) Organ Failure Assessment may serve such a purpose [20, 31]; however, initial data suggest that the sensitivity of this tool for mortality is particularly poor in the ED population [29].

Other differences between the groups in our cohort may provide insight into the factors that clinicians use when deciding on disposition. Unsurprisingly, patients with higher severity of illness at the time of ED assessment (as per APACHE II score) were more likely to be admitted to the ICU. None of the patients admitted to the ward or sent home met Sepsis-3 clinical criteria for septic shock. Given existing constraints in the availability of resources, it is understandable that patients admitted to the ICU are largely those requiring critical care interventions (such as mechanical ventilation and vasoactive medications) at the time of ICU assessment. However, there is important evidence demonstrating that ICU admission of “marginal” patients with infection (those whose ICU admission is seen as discretionary and who may be adequately managed on the ward) is associated with lower mortality than of those admitted to the wards [32]. Furthermore, we found that patients admitted directly to the ICU had higher serum lactate. Elevated ED serum lactate is known to be highly specific for mortality in admitted ED patients, but its sensitivity is poor [33, 34]. In addition to lactate, patients admitted directly to the ICU also had biochemical evidence of organ dysfunction, such as elevated creatinine and urea. Therefore, a prognostic tool such as the Sequential (Sepsis-related) Organ Failure Assessment, which includes these laboratory results, may seem attractive; however, its accuracy for ED patients with severe sepsis was inferior to its accuracy in ICU patients [18]. Taken together, our findings highlight the need to consider ICU admission in patients with high risk of future deterioration and not just those immediately requiring critical care interventions. Limitations in resources preclude the ability to admit all patients at risk of future deterioration to the ICU, but our work strongly suggests that the creation of new clinical decision instruments for risk stratification of ED patients with infection may be helpful in optimizing disposition. Furthermore, use of electronic screening tools on hospital wards may be useful in identifying patients with early clinical evidence of deterioration, who may otherwise be missed [35].

We found that patients with direct ICU admission from the ED had lower overall costs. This is likely due in large part to the shorter ICU LOS seen in these patients, which is a particularly large driver of hospital costs [36, 37]. However, cost per day was lowest among patients directly admitted to the ICU, suggesting that factors independent of ICU LOS contribute to the differences in total hospital cost. It is possible that the increased cost among these patients may be a result of illness severity, which has been suggested as a possible contributor to cost variability seen in the care of sepsis patients [38]. Furthermore, costs for ED patients with sepsis with delayed ICU disposition may be higher because of the increased mortality seen in this population. Higher mortality has been linked to higher costs [39], and it is believed to be due to the increased number of investigations and interventions in severely ill patients in the period immediately preceding death [40], as well as to the costs related to end-of-life care in the ICU [41, 42]. Understanding these hospital-wide costs among patients with delayed ICU disposition demonstrates another important administrative outcome that can potentially be improved through optimization of disposition.

Although our study demonstrates novel findings in an at-risk patient population (particularly including patients who are discharged to home), there are several limitations affecting the generalizability of our results. The most important limitation of this study is the use of ICD-CA codes for case identification, which can create misclassification bias. This is often inherent to studies involving such large databases, where diagnosis codes are used for identification [43]. We attempted to minimize misclassification by confirming diagnosis through blinded chart review. Unfortunately, we were able to review only the charts of patients with an ICD-CA code for suspected infection and not for patients with other diagnoses who may have had suspected infection. Furthermore, because we retrospectively analyzed the data collected within our registry, there were some important variables that were not captured. First, we can only speculate on reasons for disposition. In actuality, decisions related to disposition are complex and multifactorial, and often shared decision-making between clinicians and patients can influence the eventual decision. Additionally, we did not have data on patients presenting to the ED with suspected infection who were discharged to home or to the wards but did not deteriorate and require the ICU, nor did we have data on patients admitted to the wards who died prior to ICU admission. Although such an analysis was outside the scope of the current study, these patients would provide important corollary information in determining which factors are predictive of future deterioration in ED patients not directly admitted to the ICU. Furthermore, with regard to the patients who were discharged to home, we did not have data related to those patients who may have died at home. Finally, although our data were gathered from two hospitals, they exist within the same city and health network. Given the known regional variation in ICU admission practices, our findings may not be generalizable to all practice settings.

## Conclusions

We found that ED patients with suspected infection and direct ICU disposition demonstrated an association with better outcomes and lower overall costs than patients initially admitted to the floor or discharged to home. Our findings highlight the difficulty associated with predicting future short-term deterioration in patients with suspected infection in the ED, and they underscore the need for ED-specific risk stratification tools in this patient population.

## Additional files

Additional file 1: International Classification of Diseases, Tenth Revision, Canada, codes (ICD-10-CA codes). ICD-10-CA codes used for classification of suspected infection. (DOCX 92 kb)

Additional file 2: Multivariable logistic regression analysis. Multivariable logistic regression analysis of factors associated with in-hospital mortality (*n* = 657). Hosmer-Lemeshow test = 0.121. (DOCX 68 kb)

Additional file 3: Multivariable generalized linear models for total costs incurred. Multivariable generalized linear models depicting impact of variables on overall costs. (DOCX 68 kb)

## Abbreviations

ANOVA: Analysis of variance
APACHE II: Acute Physiology and Chronic Health Evaluation II
ED: Emergency department
ICD-10: International Classification of Diseases, Tenth Revision
ICU: Intensive care unit
IQR: Interquartile range
LOS: Length of stay
Sepsis-3: Third International Consensus Definitions for Sepsis and Septic Shock
SIRS: Systemic inflammatory response syndrome
