# Supplementary material for: Emergency Department disposition decisions and associated mortality and costs in ICU patients with suspected infection
Source: Crit Care. 2018 Jul 6;22:172. doi: 10.1186/s13054-018-2096-8 (PMC6034286; doi:10.1186/s13054-018-2096-8)
Supplement: Supplementary file 1 — International Classification of Diseases, Tenth Revision, Canada, codes (ICD-10-CA codes). ICD-10-CA codes used for classification of suspected infection. (DOCX 92 kb) [file 13054_2018_2096_MOESM1_ESM.docx]

***Supplemental Table 1*:** International Classification of Diseases codes (ICD-10-CA codes)

| **Included** | |
| --- | --- |
| **Disease Category** | **ICD-10-CA codes** |
| Sepsis | A02.1, A02.2, A21.7, A22.7, A26.7, A28.2, A32.7, A39.2-, A39.4, A40 (including all sub-codes), A41 (including all sub-codes), A42.7, A48.3, B00.7, B37.7, G08.0, K91.44, O75.3, O85, T81.4, |
| SIRS with Infection | R65.0 |
| Central Nervous System | G00 (including all sub-codes), G01 (including all sub-codes), G02 (including all sub-codes), G03 (including all sub-codes), G04 (including all sub-codes), G05 (including all sub-codes), G06 (including all sub-codes), G07, G08 |
| Pneumonia (without sepsis) | B59, B96.1, J11.0, J12 (including all sub-codes), J13, J14, J15 (including all sub-codes), J16 (including all sub-codes), J17 (including all sub-codes), J18 (including all sub-codes), P23.1, |
| Genitourinary infection | A54 (including all sub-codes), A56.0, N29.0, N29.1, N30 (including all sub-codes), N34 (including all sub-codes), N37.0, N39.0, N41.2, N45 (including all sub-codes), N48.20, N48.21, N49.3, N49.9, N70 (including all sub-codes), N73 (including all sub-codes), N74 (including all sub-codes), |
| Gastrointestinal infection | A09 (including all sub-codes), K35-K38, K61 (including all sub-codes), K63.0, K65 (including all sub-codes), K67 (including all sub-codes), K75.0, K80 (including all sub-codes), K81 (including all sub-codes) |
| Skin and Soft Tissue Infection | L00-L08 (including all sub-codes) |
