# Supplementary material for: Emergency Department disposition decisions and associated mortality and costs in ICU patients with suspected infection
Source: Crit Care. 2018 Jul 6;22:172. doi: 10.1186/s13054-018-2096-8 (PMC6034286; doi:10.1186/s13054-018-2096-8)
Supplement: Supplementary file 2 — Multivariable logistic regression analysis. Multivariable logistic regression analysis of factors associated with in-hospital mortality (n = 657). Hosmer-Lemeshow test = 0.121. (DOCX 68 kb) [file 13054_2018_2096_MOESM2_ESM.docx]

***Supplemental Table 2*:** Multivariable generalized linear models for total costs incurred

| **Variables** | **Total Costs** | | | |
| --- | --- | --- | --- | --- |
|  | **ß** | **95% Confidence Interval** | | ***P Value*** |
|  |  | **Lower** | **Upper** |  |
| **Age (years)** |  |  |  |  |
| < 50 | 0.094 | -0.117 | 0.305 | 0.38 |
| 50-59 | 0.121 | -0.076 | 0.319 | 0.23 |
| 60-69 | 0.00 | - | - | - |
| 70-79 | -0.036 | -0.228 | 0.156 | 0.72 |
| 80-89 | -0.105 | -0.324 | 0.113 | 0.34 |
| ≥ 90 | -0.748 | -1.245 | -0.252 | **0.02** |
| **Sex** |  |  |  |  |
| Male | 0.00 | - | - | - |
| Female | 0.003 | -0.131 | 0.136 | 0.97 |
| **Disposition from ED** |  |  |  |  |
| Intensive Care Unit | -1.398 | -1.542 | -1.255 | **<0.001** |
| Ward | 0.00 | - | - | - |
| Home | -0.365 | -0.593 | -0.138 | **<0.01** |
| **Elixhauser Comorbidity Score** | .002 | -0.008 | 0.013 | 0.68 |

**Supplemental Table 2:** Abbreviations: ED, Emergency Department.
